# Obesogen Holdover: Prenatal Exposure Predicts Cardiometabolic Risk Factors in Childhood

**DOI:** 10.1289/ehp.123-A265

**Published:** 2015-10-01

**Authors:** Lindsey Konkel

**Affiliations:** Lindsey Konkel is a New Jersey–based journalist who reports on science, health, and the environment.

Disruption of the metabolic system during critical windows of development, including the prenatal period, may predispose individuals to obesity and related diseases later in life.^1^^,^^2^ Certain chemicals, including some persistent organic pollutants (POPs), may mimic or block the actions of hormones involved in the development of fat tissue and energy homeostasis in animals and humans; these chemicals are known as obesogens.^3^ In this issue of *EHP*, researchers examine the link between prenatal exposure to three kinds of POPs and cardiometabolic risk factors in young children.^4^

Greece has one of the highest percentages of childhood obesity in the European Union.^5^ Traditional risk factors such as genetic predisposition, food consumption, and exercise don’t fully explain the obesity epidemic, according to lead study author Marina Vafeiadi, an environmental health researcher at the University of Crete.

**Figure:**
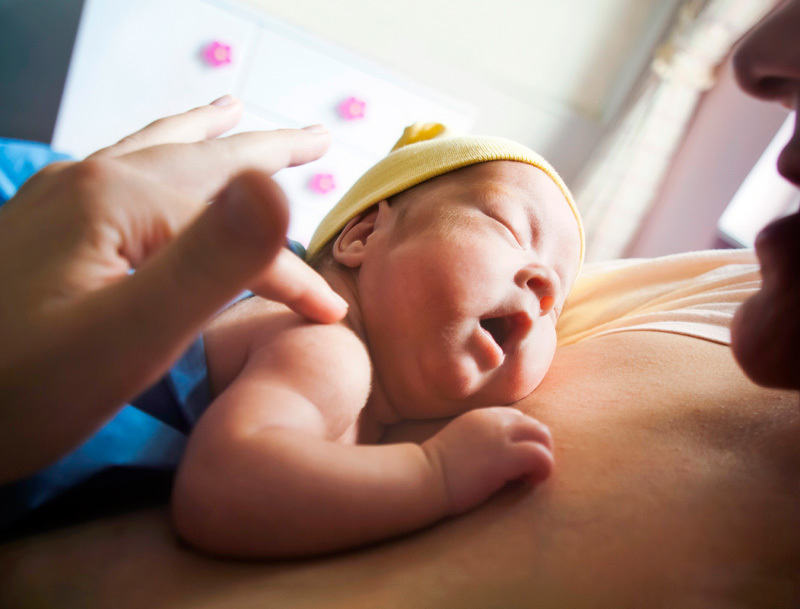
Exposure to POPs can begin long before birth, and the health effects may last a lifetime. © IvanJekic/iStockphoto

Studies in adults have linked exposures to certain POPs with cardiometabolic risk factors including high blood pressure^6^ and high blood lipids,^7^ but evidence in children is scarce.^8^ Vafeiadi says this study is the first to research the effects of prenatal POP exposure on cardiovascular traits in children.

The study included nearly 700 mother–child pairs from the Rhea cohort in Greece. The researchers measured concentrations of dichlorodiphenyldichloroethene (DDE), hexachlorobenzene (HCB), and polychlorinated biphenyls (PCBs) in blood samples taken from the mothers around the third or fourth month of pregnancy. DDE and HCB were once used widely as pesticides, while PCBs were used in many industrial processes. Although the use of these chemicals has been banned for decades in developed countries, the chemicals themselves still remain in the environment. They also bioaccumulate in the bodies of animals and humans.^9^

When the children of the current study were 4 years old, the researchers took body measurements including weight, height, waist circumference, and skinfold thickness. Other cardiometabolic risk factors measured included blood pressure and blood lipid levels. The researchers controlled for confounding factors including maternal age at delivery, mother’s pre-pregnancy body mass index (BMI) and weight gain during pregnancy, and breastfeeding duration.

At age 4, 14% of the study children were overweight. An additional 7% were obese. Their mothers had slightly lower POPs concentrations, on average, than mothers in other recent pregnancy cohorts around the world. A 10-fold increase in the mothers’ HCB concentrations was associated with higher risks of generalized and abdominal obesity in the children, greater skinfold thickness, higher systolic blood pressure, and a modest increase in child BMI *z-*score. (BMI *z-*scores are used to report obesity in children because they account for the child’s sex and exact age at assessment, in contrast to BMI, which accounts only for height and weight.) Prenatal DDE exposure was associated with higher BMI *z-*score, increased risk of abdominal obesity, and higher diastolic blood pressure. PCBs were not associated with any of the risk factors assessed.^4^

Associations between POPs exposures and cardiometabolic risk factors did not appear to differ between boys and girls. Whether child sex plays a role is of interest, says Dania Valvi, a research fellow at Harvard T.H. Chan School of Public Health. That’s because animal data^10^ and previous studies on childhood BMI ^11^^,^^12^^,^^13^ reported effect modification by sex, which Valvi says is worth exploring further in larger populations. Valvi was not involved with the current study.

“The results are pretty consistent for HCB and DDE across several measurements of adiposity, which strengthens confidence that the associations are real,” says Michele La Merrill, a toxicologist at the University of California, Davis. While it is not completely clear what the findings could mean for future health outcomes among the children, past studies have reported associations between childhood obesity and conditions such as sleep apnea, precocious puberty, adult obesity, and coronary heart disease, say La Merrill, who was not involved with the study.

The authors maintain that as Greece, the United States, and other countries around the world deal with an increasing prevalence of childhood obesity, the findings are important from a public health perspective. Knowledge of environmental risk factors could help to reverse the trend, according to Vafeiadi. “It’s important to monitor risk factors and develop interventions in a young population before [unhealthy] lifestyle choices have been established,” she says. Vafeiadi and colleagues hope next to study exposures to nonpersistent chemicals (such as bisphenol A) and combinations of suspected obesogens in relation to cardiometabolic risk factors in the Rhea cohort.
